# Recent advances in biomedical simulations: a manifesto for model engineering

**DOI:** 10.12688/f1000research.15997.1

**Published:** 2019-03-05

**Authors:** Joseph L. Hellerstein, Stanley Gu, Kiri Choi, Herbert M. Sauro

**Affiliations:** 1 eScience Institute, University of Washington, Seattle, WA, USA; 2 Department of Bioengineering, William H. Foege Building, University of Washington, Seattle, WA, Box 355061, USA

**Keywords:** Modeling, Systems Biology, Best Practice, Software Engineering

## Abstract

Biomedical simulations are widely used to understand disease, engineer cells, and model cellular processes. In this article, we explore how to improve the quality of biomedical simulations by developing simulation models using tools and practices employed in software engineering. We refer to this direction as model engineering. Not all techniques used by software engineers are directly applicable to model engineering, and so some adaptations are required. That said, we believe that simulation models can benefit from software engineering practices for requirements, design, and construction as well as from software engineering tools for version control, error checking, and testing. Here we survey current efforts to improve simulation quality and discuss promising research directions for model engineering.

## Introduction

Quantitative models are at the core of science and engineering. For example, the field of atmospheric sciences has provided increasingly accurate weather predictions over ever longer time horizons ^1^, capabilities that greatly benefit agriculture, shipping, and many leisure pursuits.

**Figure 1.**
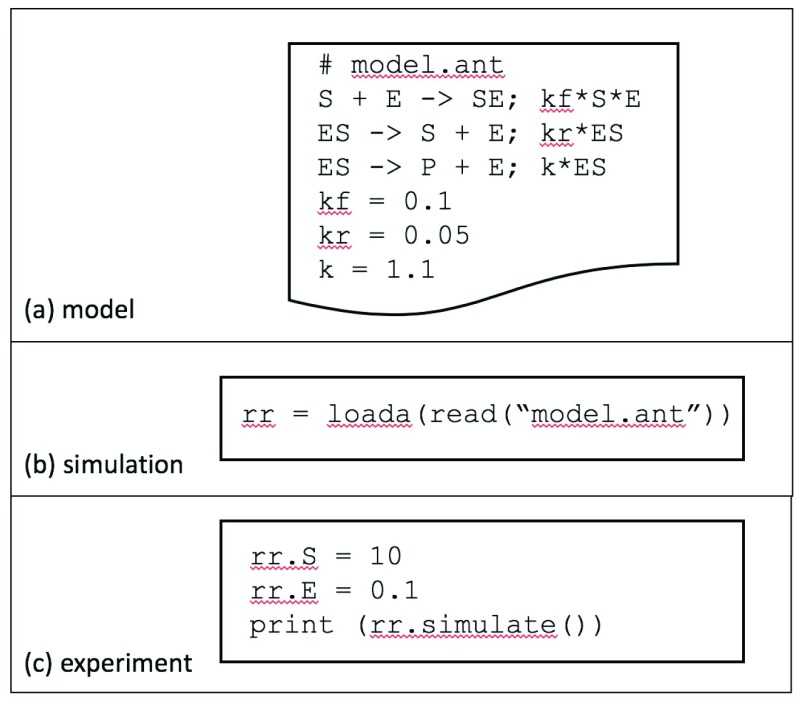
Illustration of core concepts. ( **a**) A model of enzyme kinetics is expressed in the Antimony Language. ( **b**) The simulation object rr is created from the model in ( **a**). ( **c**) The experiment uses the simulation object to specify initial values for S and E, runs the simulation, and prints the results.

A simulation model (hereafter, just model) quantifies relationships between input variables and output variables using differential equations, Boolean expressions, Petri Nets, and/or other techniques ^2– 4^. Figure 1(a) contains a model of enzyme kinetics expressed in the Antimony ^5^ language. A simulation is a software implementation of a model. Sometimes, the model is embedded within the computer codes such as Python or MATLAB. However, a recommended practice is to have models in a computer-readable representation from which simulation codes are derived automatically, as in Figure 1(b). An experiment is an execution of simulation codes for values of simulation parameters, such as specifying initial concentrations for S and E in Figure 1(c). A biomedical modeling project typically constructs models, conducts validation experiments (e.g. by comparing simulation results with empirical data), and runs experiments that make predictions of biological significance.

Current practice in biomedical simulations has numerous shortcomings that greatly limit the value provided to translational medicine and related areas. One common complaint is that the results of published biomedical models are difficult to reproduce and so are not widely used or trusted. Another concern is that published models are difficult to understand, especially to ascertain the biology being modeled. Furthermore, existing models are not constructed with an eye towards reuse, and so it is challenging to use existing models as building blocks to construct larger models, such as whole-cell models.

This article reviews current practice in biomedical simulations, especially how models are specified, designed, and constructed. Our central observation is that the issues raised in the above critiques of biomedical simulations have close analogues in software projects, an observation made by others as well (e.g. 6) This motivates a research initiative that we refer to as model engineering: how biomedical models can be improved by using best practices and tools adapted from software engineering.

The impact of model engineering will in part depend on addressing two challenges. First, some central principles of software engineering are difficult to apply to biomedical models, and so it may be impossible to use software techniques that rely on these principles. For example, reuse of software is often achieved by creating modules that hide the internals of their operation. However, as others have noted ^7^, information hiding is problematic for biomedical models because having models work in combination requires an understanding of all chemical species and reactions that they model. Second, some of the benefits of model engineering may require changes in how modeling is done, such as using meaningful names for chemical species. Such behavioral changes can be difficult to achieve.

## Critiques of current practice

### Reproducibility

Scientific inquiry is largely based on conducting experiments. An experiment is reproducible if it can be conducted by many researchers, possibly with modest variations, and similar results are obtained. Reproducibility is at the heart of scientific progress.

Unfortunately, many scientific papers contain results that cannot be reproduced. For example, a survey of 1,500 scientists found that over 70% had been unsuccessful in reproducing published results ^8^. A tangible impact is an increased number of retracted papers and failed clinical trials ^9^.

Ensuring reproducibility seems manageable (even trivial) for computational studies if the same simulations are run repeatedly in the same computing environment. Unfortunately, reproducibility of simulation experiments is often problematic. The authors of 9 cite issues such as (1) the computer codes may not be runnable in environments other than those used in the published work (e.g. a special purpose supercomputer) and (2) the specifics of the computational environment may not be documented in sufficient detail and thus cannot be reconstructed by others.

Much work has addressed improving the reproducibility of biomedical simulations. The most well-known examples are standardized formats that ensure reproducibility and exchangeability of simulation studies. Standards such as Systems Biology Markup Language (SBML) ^10^ and CellML ^11^ provide community-approved formats for sharing simulation models, and public repositories with models in these formats are available ^12^. The Simulation Experiment Description Markup Language (SEDML) ^13^ provides a standard for describing simulation experiments. More broadly, the MIRIAM system ^14^ establishes standards for the information that must be contained in a model so that it is reproducible, and the Systems Biology Ontology ^14^ provides a way to specify what is being modeled in a common way.

Software engineering has several techniques that can complement current efforts to create reproducible biomedical simulations. Consider a situation in which an SBML model of modest complexity (e.g. a hundred or more reactions) does not reproduce a published result. We are faced with the problem of determining why the model is not reproducible. A similar situation arises when a software package is installed and fails to work properly.

Several techniques are used by software engineers to address this problem. First, the best practices for building software include the development of unit tests that assess the correctness of fine-grain parts (units) of a software package. By running unit tests, we can isolate the cause of the package failure. Others have noted the potential value of unit tests for biomedical models ^15^. Second, software, like biomedical models, is easily changed. Software engineers use version control ^16^ to identify when a change caused unit tests to fail, which is akin to a loss of reproducibility. Here too, others have recommended the use of version control for biomedical models ^17^. Last, it may be that a software failure is not the result of a problem with the new package *per se*. Rather, the failure may be due to conflicts between the new package and other software installed on the same computer (e.g. inconsistent versions of Python). Software engineers use package managers to handle the consistency of software versions. As biomedical models grow in size, it is likely that there will be attempts to combine models that conflict in some way. A package manager for models may benefit biomedical simulations as reuse becomes common.

### Readability

Model readability is about the ability of humans to read and understand a model. Readability is often overlooked, and this can have serious consequences. For example, the Therac-25 radiation therapy machine was involved in at least six accidents in which patients were given massive overdoses, resulting in several deaths because of legacy code that was poorly understood ^18^.

Much work has been done to remove ambiguity in biomedical models. For example, annotations (e.g. 19) can be applied to detect that two names refer to the same chemical species, and annotations can be used to clarify which pathways are being modeled.

Another contribution to readability is rule-based systems (e.g. 20). Rules can improve human understanding by using hierarchies to organize details of models, such as an organization by cell structures.

Still other contributions to model readability are tools that convert computer encodings into a human-readable equivalent. Two examples are converting SBML into LaTex ^21^ and translating SED-ML into readable text ^22^.

Software engineering addresses readability in a much more comprehensive way (e.g., 23). This is in part because of the commercial importance of software to companies such as Google, Facebook, and Microsoft. Another reason for the focus on readability is the scale and complexity of software. Open source projects such as Firefox and Linux have several million lines of code, and human understanding of these codes is essential for fixing errors and adding features. In contrast, a typical model in BioModels has under 100 reactions. Even very large models such as whole-cell models ^24^ have a far lower complexity than popular open source software projects. However, to develop whole-tissue, whole-organ, and even whole-organism models, the complexity of biomedical models will grow dramatically. We believe that the experience with readability of software offers guidance for the readability of biomedical models.

One insight from software relates to a seemingly mundane consideration––the choice of variable names. The biomedical modeling community has devoted much effort to annotations (e.g. identifying the chemical species associated with a name) but not to the choice of names *per se*.

**Figure 2.**
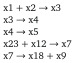
Snippets of a model of the MAPK cascade that uses variable names that impair readability.

**Figure 3.**
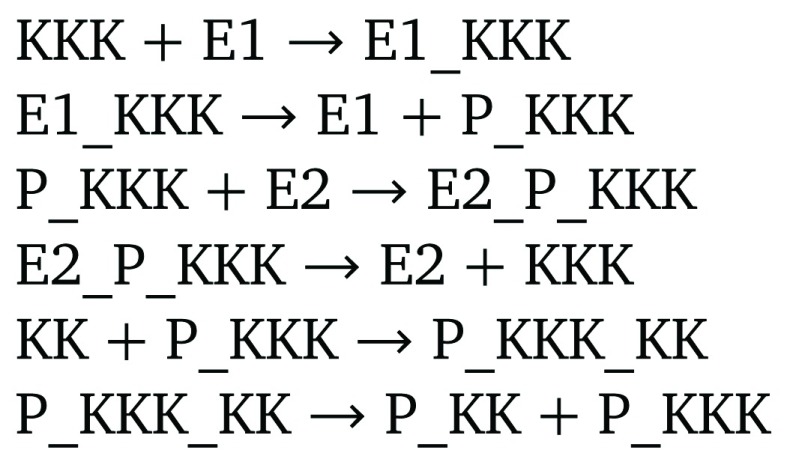
Snippets of a model of the MAPK cascade that uses variable names that promote readability.

We illustrate the importance of the choice of species name to readability. Consider two models of the MAPK cascade that are curated in the BioModels repository ^12^ according to the MIRIAM standard. Figure 2 displays a snippet of model BIOMD0000000019. This model uses numbered values of the letter x to denote different chemical species. Although this is reasonable for a computer representation of the chemical species, human readability is impaired. Now, consider BIOMD0000000010 and the snippet of this model in Figure 3. We see that the names are chosen in a systematic way that promotes readability. Specifically, KK denotes the MAPK kinase, KKK denotes the MAPK kinase kinase, E1 and E2 are enzymes, and P_X indicates the phosphorylation of the molecule X.

Because of the importance of readability in software engineering, there are style guides for every major computer language (e.g. see 25 for Python style) that dictate details such as how to name variables, structure codes, and even the location of white spaces to improve human readability. In recent years, the scope of readability of software codes has been extended to an idea called literate programming that mixes human written text with computer codes ^26^. Literate programming motivated the development of tools such as Jupyter Notebooks ^27^, R markdown ^28^, and the Tellurium Notebook ^29^. These tools are a kind of living laboratory notebook that describes the assumptions made, procedures used, and outcomes observed. Literate programming provides the additional benefit of being able to easily change modeling assumptions and/or procedures and then re-run the simulation.

### Reuse

A component is reusable if it can be readily embedded into many different systems. The multi-trillion-dollar software industry is largely built on reusable components. Waltemath *et al.* ^15^ and Goldberg *et al.* ^6^ argue for building models so that they can be reused, but both observe that in current practice it is rare for a model to be embedded as-is into another model.

At first glance, it seems surprising that models are rarely reused, since there are hundreds of models in each of the BioModels and CellML repositories that are in computer-readable standard formats. Furthermore, the use of constructs such as the Antimony ^5^ model statement provides a convenient way to reuse model subparts. Given this, why not approach reuse as is done in software engineering?

To date, model reuse has largely focused on modularization––organizing codes as a collection of fairly coarse-grain components called modules; modules are the unit of reuse. This approach is widely used in software systems and largely rests on the principle of information hiding. That is, a component exposes an interface that describes what inputs it takes and what outputs it produces. How the outputs are produced is hidden. Software components that abide by the principle of information hiding and have no side-effects can be combined without concerns of unintended interactions with other components, since the component internals are hidden from one another.

Unfortunately, information hiding can be difficult to achieve when embedding biomedical models, at least if the embedded models describe pathways in the same biological compartment. Consider how to build an integrated model of the glycolysis and pentose phosphate pathways from two separately developed models of each pathway. The problem is that to correctly simulate the pathways in combination, the integrated model must take into account chemical species that are in common to both (e.g. fructose 6-phosphate).

Annotations are an important part of solving this problem by allowing the identification of identical chemical species in different models. Indeed, the use of annotations with appropriate software tools can greatly facilitate the detection of situations in which the same chemical species is present in multiple modules ^7^.

We observe that annotations alone do not solve the problem of model reuse because reuse has integration considerations such as (a) defining equivalences between chemical species in different submodels, (b) adding side-reactions to handle interactions between intermediate species in submodels, and (c) handling reactions that are duplicated across submodels. Fortunately, software engineering has developed a number of ways to structure codes that enable a more fine-grain integration of components than is possible with modularization. We further note that it is important to record the changes made when reusing a model and the reasons for making these changes to better understand differences in experimental results.

## Model engineering

The previous section discusses shortcomings in current practice for biomedical simulations in reproducibility, readability, and reuse. Concerns in these areas are very similar to challenges faced by the software industry with creating, evolving, and maintaining large software systems. Indeed, the huge success of the software industry over the last 50 years is in many ways a result of providing solutions for reproducibility, readability, and reusability. In this section, we explore the extent to which the tools and practices of software engineering can improve the quality of biomedical simulations.

Software engineering can be described as “the application of a systematic, disciplined, quantifiable approach to the development, operation, and maintenance of software” ^16^. Practitioners often segment software projects into phases referred to as the software life cycle.

• The requirements phase defines what is to be built.
• The design phase specifies the components used and how they interact to address the requirements.
• The construction phase implements the design.

Even though phases are listed in sequence, phases are not strictly linear. Rather, software development is an iterative process, and there is considerable variability in the steps used from project to project ^30, 31^. That said, there are activities that are common to a diverse set of projects. As a result, a variety of tools have been developed to aid the engineering process.

To illustrate the application of software engineering to biomedical simulations, we use a running example: building a simulation that combines the glycolysis pathway with the pentose phosphate pathway (PPP) ^7, 32^.

### Requirements

A requirement is a description of what a system should do. In our running example, the requirement is to create an integrated model of the glycolysis and PPP pathways by combining separate models of each pathway.

Requirements can be further refined by describing use cases. In software, a use case describes how the user interacts with the system. For simulation models, we define a use case as the information provided by running the simulation model.

Some elements of a model use case are:

• Biological process: the chemical pathways that are addressed by the model (e.g. glycolysis, MAPK cascade)
• Coverage: the parts of the biological scope that are included in the model, such as the chemical species, reactions, and regulators
• Location: the biological compartment in which the reaction takes place.

Here, we explore a limited part of software design––code structure––and the application of code structuring techniques to improving biomedical models.

Explicit articulation of use cases can address some of the critiques of current practice for biomedical simulations. Use cases provide a clear statement of the purpose of the model. This enhances readability in the same way that it is easier to read a book with a familiar plot. Further, use cases increase model reuse by allowing modelers to identify existing models that are similar to what is required for a new modeling project.

We see a number of research directions related to the requirements phase of model engineering. First, there is considerable benefit in developing a formal vocabulary for expressing use cases for biomedical simulations. A good foundation exists already––the BioModels ^12^ advanced search capability that allows for a search by categories within the gene ontology. More work is needed, however, to flesh out the elements of a model use case and to provide a controlled vocabulary for considerations such as coverage. A second research direction is to develop algorithms for searching model repositories based on elements of use cases and effectively present results for complex searches.

### Design

A design specifies the components used and how they interact to address the requirements. We use the term component to mean something more general than a module. For example, a component of a biomedical model might be as fine grain as a reaction or even the kinetics of a reaction. Here, we explore a limited part of software design––code structure––and the application of code structuring techniques to improving biomedical models.

As noted earlier, a common way to structure software is modularization, in which code is organized into relatively independent pieces called modules. Each module performs a limited set of functions, and modules do not expose the internals of how their functions are accomplished.

We consider a modular design for the integrated glycolysis and PPP pathways. A first step is to find existing models for each pathway. To this end, we search BioModels using the controlled vocabularies that are part of the MIRIAM standard. Using the glycolysis process annotation from the gene ontology, GO:0006096, we find 35 models. Similarly, we search for models labeled with the pentose phosphate shunt, GO:0006098. We obtain 11 results.

**Figure 4.**
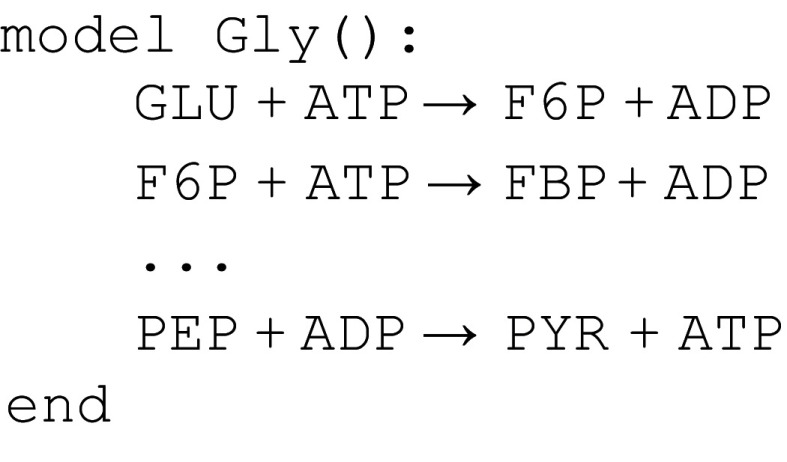
Snippet of the glycolysis model in 33.

**Figure 5.**
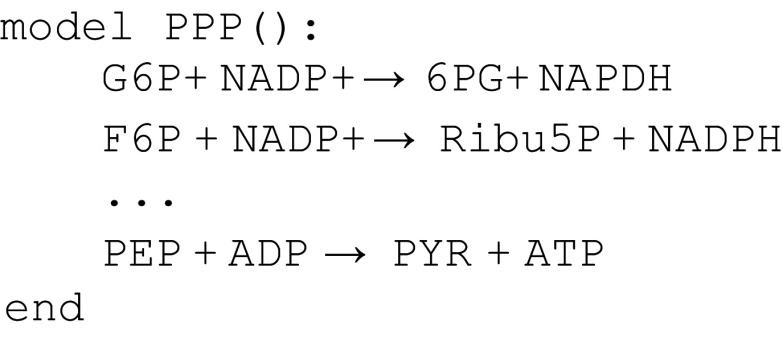
Snippet of the pentose phosphate pathway model in 34.

We create a glycolysis module using Nielsen *et al.* ^33^ and a PPP module using Chassagnole *et al.* ^34^. Figure 4 contains a snippet of the glycolysis module using Antimony syntax, and Figure 5 does the same for PPP.

**Figure 6.**
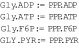
Some of the equivalences required between Gly and PPP.

We see that both modules reference the names ADP, ATP, G6P, and F6P. Although it may be obvious to the reader that the intent is to refer to the same chemical species, the semantics of the model statement are that the names in Figure 4 refer to different chemical species from the same names in Figure 5. One way to resolve this problem is to make explicit equivalences between chemical species ^7^. Figure 6 demonstrates how to accomplish this in Antimony.

Making specific equivalences is burdensome if there are many models with chemical species in common. Similar problems arise in software as well. The software solution is to use name scoping ^35^. A scope is the codes for which a name refers to the same entity. For the running example, the appropriate scope is a biological compartment––in this case, the cytoplasm. Using names with a compartment scope eliminates the need for making explicit equivalences as in Figure 6. Furthermore, with name scoping and consistent use of names, name equivalences are made automatically when a new model is added.

A key shortcoming of modularization is that a module must be either used in its entirety or not used at all. Often, we need greater selectivity as to which model elements are reused. One way to provide this is with object oriented programming ^36^. Object-oriented approaches structure shared codes into one or more "parent classes" that are "inherited" to create specialized modules. This turns out to be useful in the running example. Chassagnole *et al*. model parts of the glycolytic pathway as well as PPP. Using object hierarchies, we can reorganize the models so that the glycolysis parts of Chassagnole *et al*. are in a parent class from which both the reorganized Chassagnole model and a refactored version of the Gly inherit. Object hierarchies are used in areas of systems biology such as the Functional Model of Anatomy ^37^. However, we are unaware of the use of object hierarchies in community standards for reusable models.

Another powerful technique for structuring software is aspect-oriented programming (AOP) ^38^, in which code is inserted for every occurrence of a pattern. For example, in software AOP can be used to count function calls (e.g. as part of a profiler); the pattern is a function definition, and the inserted code is a call to an instrumentation function. AOP has the potential to simplify biomedical models and increase reuse. Consider the specification of reaction kinetics. AOP (in combination with consistent naming of enzymes) makes it easy to specify Michaelis-Menten kinetics for every enzyme-catalyzed reaction and specify mass balance kinetics if the reaction is not enzyme catalyzed. In this case, the pattern is the presence or absence of an enzyme, and the inserted element is the kinetics law.

### Construction

Construction is about implementing a design. This involves implementing new components (coding), revising components, and detecting errors in components. These tasks have direct analogs in the construction of biomedical models.

Coding a biomedical model can be done in a general purpose programming language such as Python, Java, and FORTRAN. However, there is considerable benefit to using domain-specific languages (DSLs) that are tailored to biomedical modeling. Examples of DSLs include Antimony ^5^ and Jarnac ^39^ as well as interchange formats, such as SBML ^10^ and CellML ^11^.

As we have emphasized throughout, a key reason for the success of the software industry is the focus on reuse. When an application incorporates software from many sources, the embedded software is referred to as dependencies. The embedding application wants to incorporate future changes to embedded software that fixes errors and adds features. However, there may be changes in dependencies that "break" the application because embedded codes work differently. Detecting and resolving breaking changes is central to dependency management. In software systems, this is typically handled by a package manager ^16^ that can list, add, update, and remove dependencies. Often, dependencies must be analyzed transitively, since a dependency may itself have dependencies that need to be managed (e.g. the operating system version on which the web browser depends).

If biomedical models are going to be reused, then dependency management must be addressed. For example, changes in the intermediate species used in an embedded model can affect the efficiency and correctness of the embedding model. In the running example, we build a model for the glycolysis and PPP pathways by combining separate models of each pathway. Dependency management is required to take advantage of future bug fixes and enhancements in these embedded models. However, we do not want to incorporate changes that violate the assumptions of our combined model (e.g. related to the metabolites present).

Software is testable if it is structured so that components can be tested independently and in combination. The former are referred to as unit tests and the latter as system tests. A test has two parts: (1) the invocation of the code being tested and (2) a comparison of the outputs returned to their expected values Waltemath *et al.* ^15^ note the importance of systematic testing in building whole-cell models.

Significant effort is required to write good tests. Indeed, writing tests can be as demanding as writing the code being tested. Even so, the experience from the software engineering is that time devoted to writing tests is well worth the investment because of the dramatic improvement in software quality. The SciUnit project ^40^ provides a way to write tests for any simulation output. A test specifies simulation outputs and a Boolean valued function that is evaluated on those outputs.

Contrary to its name, SciUnit provides system tests, not unit tests, because a full model is being tested; unit tests are more fine grain. For example, a unit might be an individual reaction, and a unit test might evaluate if the reaction kinetics provide bounded reaction rates. We see the development of unit tests for models as an exciting research direction.

Testing detects errors by running experiments. This is referred to as dynamic analysis. Dynamic analysis has the benefit of being able to check a broad range of errors, but it is time consuming and often non-trivial to resolve the errors that are discovered. Software engineering complements dynamic analysis with static analysis of codes. For example, tools called linters examine source codes for errors such as referencing a variable before it is assigned a value ^41^.

Static analysis can be applied to simulation models. For example, a well-formed reaction should preserve mass balance. That is, the sum of the masses of the reactants should equal the sum of the masses of the products. Mass balance is easy to check if chemical species have annotations that provide machine-readable chemical formulas. Unfortunately, in current practice, it is unusual to have such detailed specifications of the chemical species.

Fortunately, static checking for mass balance is possible even without annotations, although for a somewhat weaker condition called stoichiometric inconsistency. We illustrate this by example. Suppose we have the following reactions:

A + B → C

B → C

The first reaction implies that the mass of C is greater than the mass of B. The second reaction implies that B and C have the same mass. That is, these reactions have a stoichiometric inconsistency. Stoichiometric inconsistencies can be detected automatically using techniques as in 42, and this can be done without annotations.

Other potential errors that can be detected statically include duplicate reactions, dubious kinetics expressions (e.g. no reactant is present in the kinetics expression), and superfluous chemical species that always have a concentration of zero. A very promising research direction is to develop linters for biomedical models that detect a wide range of static errors and report on poor modeling practices such as poorly chosen names for chemical species.

## Conclusions

Common critiques of biomedical simulations point to issues with reproducibility, readability, and reuse. Many have proposed addressing these critiques by making use of elements of software engineering practice. Herein, we argue for a broad research agenda, called model engineering, that systematically adapts techniques from software engineering to building models of biomedical systems.

We organize model engineering along the lines of software engineering, with separate phases for model requirements, design, and construction. For requirements, we advocate the development of use cases that describe the information provided by a model. For design, we focus on model reuse, especially two techniques from software engineering: name scoping and code structuring (e.g. modularization, object hierarchies, and aspect oriented programming). For construction, we emphasize (a) domain-specific languages (DSLs), (b) dependency management that assists with handling embedded models, (c) testing to detect defects from running models, and (d) linters that do static error checking. The benefits of these directions are summarized in Table 1.

**Table 1.** Benefits of model engineering. The benefits are improvements in reproducibility, readability, and reuse. The benefits are organized by lifecycle phase: requirements (R), design (D), and construction (C).

SWE Practice	Reproducible	Readable	Reuse
R: Use Cases	✓		✓
D: Name scope		✓	✓
D: Code Structure		✓	✓
C: Domain Specific Languages		✓	✓
C: Dependency Management	✓		✓
C: Unit tests	✓		✓
C: Linters		✓	✓

The impact of model engineering depends in part on the extent to which it is possible to adapt principles from software engineering to biomedical models. For example, the software engineering principle of information hiding is unlikely to be effective for biomedical models (at least for combining models whose scope is the same biological compartment). This is problematic in terms of using modularization as a way to construct reusable models. Fortunately, software engineering provides many techniques other than modularization to structure codes for reuse (e.g. object-oriented programming and AOP). It may be that these alternative techniques are more effective for structuring biomedical models for reuse.

A second constraint on the impact of model engineering is that realizing some of its benefits requires changes in current modeling practice. For example, one of our recommendations is that modelers use meaningful names to make models more readable. Such behavioral changes can be difficult to achieve.

Model engineering can be advanced in many ways. Considerable impact is possible by working directly with journals and funding agencies to argue for the inclusion of model engineering in paper submissions and funding proposals by requiring discussions of model requirements, designs, and construction. A second approach is to advocate for model engineering directly with the modeling community through publications and workshops. Last, some leverage may be possible by working with the computer science community where there is great interest in the problems addressed by model engineering and the use of software engineering to solve these problems (e.g. 43).
